# Nozzle-Shaped Electrode Configuration for Dielectrophoretic 3D-Focusing of Microparticles

**DOI:** 10.3390/mi10090585

**Published:** 2019-08-31

**Authors:** Salini Krishna, Fadi Alnaimat, Bobby Mathew

**Affiliations:** 1 Mechanical Engineering Department, United Arab Emirates University, Al Ain, UAE; 2 National Water Center, United Arab Emirates University, Al Ain, UAE

**Keywords:** dielectrophoresis, focusing, microchannel, microfluidics, microparticles, modeling

## Abstract

An experimentally validated mathematical model of a microfluidic device with nozzle-shaped electrode configuration for realizing dielectrophoresis based 3D-focusing is presented in the article. Two right-triangle shaped electrodes on the top and bottom surfaces make up the nozzle-shaped electrode configuration. The mathematical model consists of equations describing the motion of microparticles as well as profiles of electric potential, electric field, and fluid flow inside the microchannel. The influence of forces associated with inertia, gravity, drag, virtual mass, dielectrophoresis, and buoyancy are taken into account in the model. The performance of the microfluidic device is quantified in terms of horizontal and vertical focusing parameters. The influence of operating parameters, such as applied electric potential and volumetric flow rate, as well as geometric parameters, such as electrode dimensions and microchannel dimensions, are analyzed using the model. The performance of the microfluidic device enhances with an increase in applied electric potential and reduction in volumetric flow rate. Additionally, the performance of the microfluidic device improves with reduction in microchannel height and increase in microparticle radius while degrading with increase in reduction in electrode length and width. The model is of great benefit as it allows for generating working designs of the proposed microfluidic device with the desired performance metrics.

## 1. Introduction

Microfluidic devices employ channels with hydraulic diameters smaller than 1 mm and this brings about certain advantages such as low sample and reagent requirement, small footprint, portability, and low power consumption [1,2]. Microfluidic devices are used for several applications including type- and size-based separation of microparticles [3]. For achieving separation, it is important to investigate the characteristics of each microparticle and subsequently move it to the appropriate group. This requires arranging randomly dispersed microparticles in an orderly fashion. Conventional biomedical devices such as flow cytometers (separating and counting of cells) and Coulter counters (counting of cells) are being miniaturized to avail several of the aforementioned benefits [4,5]. For these devices to function efficiently, it is important that cells pass through the sensing zone of the same individually, i.e., arrangement of randomly dispersed cells in an orderly fashion. Arranging of randomly dispersed microparticles is referred to as focusing [6]. Three-dimensional (3D) focusing refers to arranging randomly ordered microparticles in a single file. Several non-invasive actuation forces, including dielectrophoresis (DEP), are employed in microfluidic devices for 3D-focusing [6]. DEP refers to the movement of dielectric microparticles, suspended in an electrically conductive medium when subjected to a non-uniform electric field [7]. When the movement of the microparticle is towards the maxima of the electric field then, DEP is referred to as a positive-DEP (pDEP). On the other hand, DEP is referred to as negative-DEP (nDEP) when the movement of the microparticles is towards the minima of the electric field [7]. Employing DEP in microfluidic devices requires neither sheath flow nor specialized wafers and these represent the merits of DEP over other types of actuation forces. Additionally, DEP scales well with miniaturization, i.e., the electric field required for realizing DEP can be achieved with low voltages. The time averaged magnitude of the force associated with DEP is provided in Equation (1) [7].

(1) $$\left[\begin{array}{c} F_{DEP,x}\\ F_{DEP,y}\\ F_{DEP,z} \end{array}\right]=2\pi \varepsilon_{m}r_{p}^{3}Re\left[f_{CM}\right]\left[\begin{array}{c} \frac{\partial }{\partial x}\\ \frac{\partial }{\partial y}\\ \frac{\partial }{\partial z} \end{array}\right]E_{RMS}^{2}$$

The real part of the Clausius–Mossotti factor (*f*_CM_) is dependent on the permittivity and conductivity of the medium and microparticle as shown in Equation (2); Equation (3) presents the relationship of conductivity of microparticle to its bulk and surface conductivities [8]. The polarity of *Re*[*f_CM_*] determines whether a microparticle will experience pDEP or nDEP; when *Re*[*f_CM_*] is positive, then the microparticle will experience pDEP and if *Re*[*f_CM_*] is negative, then the microparticle will experience nDEP.

(2) $$Re\left[f_{CM}\right]=\frac{\left(\varepsilon_{p}+2\varepsilon_{m}\right)\left(\varepsilon_{p}-\varepsilon_{m}\right)+\frac{\left(\sigma_{p}+2\sigma_{m}\right)\left(\sigma_{p}-\sigma_{m}\right)}{\omega^{2}}}{{\left(\varepsilon_{p}+2\varepsilon_{m}\right)}^{2}+\frac{{\left(\sigma_{p}+2\sigma_{m}\right)}^{2}}{\omega^{2}}}$$

(3) $$\sigma_{p}=\sigma_{bulk,p}+2\frac{K_{s,p}}{r_{p}}$$

**Figure 1:**
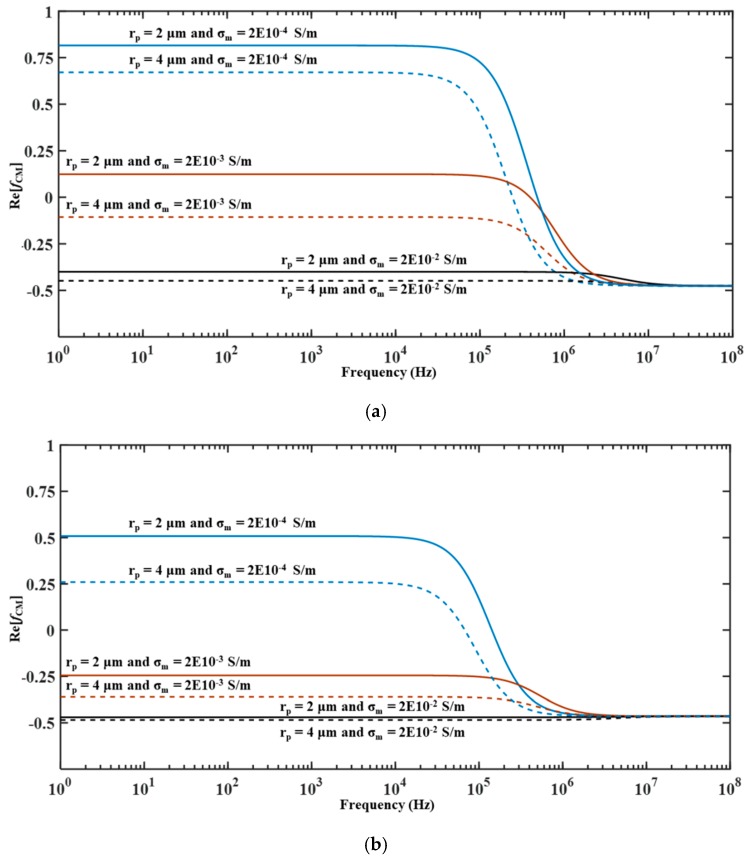
Variation of *Re*[*f_CM_*] with frequency for (**a**) polystyrene and (**b**) silica microparticles.

It can be noticed from Equation (2) that as *ω* → 0, *ε_m_* and *ε_p_* do not influence *Re*[*f_CM_*]. On the other hand, the influence of *σ*_m_ and *σ*_p_ on *Re*[*f*_CM_] is non-existent as ω → ∞. *Re*[*f_CM_*] of microparticles such as polystyrene, latex, and silica microparticles is positive and negative as *ω* →0 and ω → ∞, respectively. In most microfluidic devices, the electrodes are located near the sidewalls and it is desired to 3D focus microparticles at the center of the microchannel; for this, it is necessary to subject microparticles to nDEP which in turn requires operating the microfluidic device at high operating frequency. Figure 1 provides the variation of *Re*[*f_CM_*] with operating frequency with respect to the radius and conductivity of the medium for polystyrene (*ε_p_* = 2.55*ε_o_* F/m, *ρ_p_* = 1055 kg/m^3^, *σ_bulk,p_* = 0, and *K_s,p_* = 2.85 × 10^−9^ S) and silica microparticles (*ε_p_* = 3.8*ε*_o_ F/m, *ρ_p_* = 2000 kg/m^3^, *σ_bulk,p_* = 0, and *K_s,p_* = 0.82 × 10^−9^ S) [8]. Figure 1 reveals that these microparticles experience nDEP at high operating frequencies as mentioned earlier. Additionally, it can be noticed that these microparticles experience nDEP at high operating frequencies irrespective of the conductivity of the medium; this implies that the performance of any microfluidic device that employs nDEP for purposes of focusing is independent of the conductivity of the medium.

**Figure 2:**
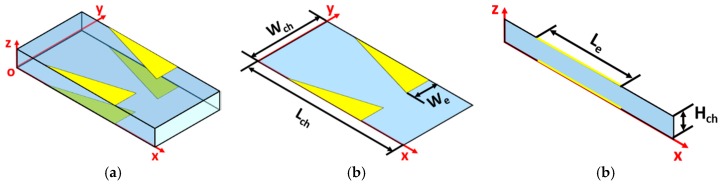
Schematic of the electrode configuration for dielectrophoresis (DEP)-based three dimensional (3D) focusing analyzed in this article (**a**) perspective view, (**b**) top view, and (**c**) side view.

Figure 2 shows the microfluidic device analyzed in this article. The electrode configuration consists of two electrodes on the top surface and two electrodes on the bottom surface; all electrodes are right-triangle shaped and each electrode on the top surface aligns with a similar electrode on the bottom surface. For this electrode configuration, the minima of the gradient of the electric field is located at the center of the microchannel. Thus, any microparticle subjected to nDEP using the electrode configuration shown in Figure 2 will be pushed towards the center of the microchannel thereby achieving 3D-focusing of microparticles; the electrode configuration functions like a nozzle with regard to the movement of microparticles.

**Figure 3:**
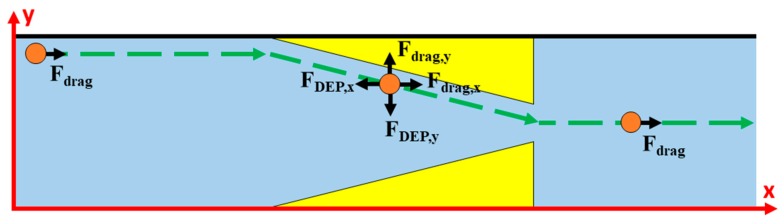
Schematic of the top-view of the trajectory of microparticle.

Figure 3 depicts the top view of the trajectory of a typical microparticle along with the prominent forces acting on it inside the microfluidic device. In the regions away from the electrodes, the prominent force acting on the microparticle is that due to drag while in the regions near the electrodes, the prominent forces acting on the microparticle are those associated with drag and nDEP. In the presence of all forces, it is important that the microparticle is subjected to nDEP force for sufficient duration to be deflected along the desired path. 

Regarding the electrode configuration shown in Figure 2, the starting width of the electrodes is zero and this allows for focusing microparticles that are originally located very close to the sidewalls; this is the primary merit of the proposed electrode configuration. Holmes et al. [9] previously showed that microfluidic device employing electrodes with non-zero starting width might not always focus microparticles that start from regions close to the sidewalls. Moreover, the chip-to-world electrical connection is easier for this electrode configuration compared with the electrode configuration consisting of multiple finite-sized interdigitated transducer (IDT) electrode pairs [10]. Additionally it is possible to apply nDEP over a greater length with the electrode configuration in Figure 1 compared with finite-sized IDT electrodes pairs. The ability to apply nDEP over a greater length allows for either handling greater throughput for a specific applied electric potential or reducing the applied electric potential for a specific throughput [10]. The proposed electrode configuration can be incorporated along with other phenomena, such as acoustophoresis, magnetophoresis, and inertial microfluidics, as required in the same microfluidic device [11,12,13].

Researchers have developed DEP based microfluidic devices for 3D-focusing of microparticles. Morgan et al. [14] modeled and built a microfluidic device, with two planar electrodes each on the top and bottom surfaces of the microchannel, for purposes of 3D-focusing. Each planar electrode has an upstream curved section and a downstream rectangular section. The four curved sections function together to direct all microparticles, by subjecting them to nDEP, to the central region of the microfluidic device between the four rectangular sections. Subsequently, the microparticles are subjected to nDEP by the rectangular section of the electrodes to 3D-focus them at the center of the microchannel. Yu et al. [15] created a microfluidic device with interdigitated transducer (IDT) electrodes, running over the entire circumference of a circular microchannel, for 3D-focusing of a microparticle. In this microfluidic device, an electric field is established between every electrode and its two neighboring electrodes. The minima of the gradient of the electric field is at the center of the microchannel due to which microparticles subjected to nDEP are focused at the center of the microchannel. Holmes et al. [9] constructed a microfluidic device with two pairs of trapezoid-shaped electrodes. Electrodes of each pair protrude into the microchannel from the sides; one electrode of a pair is located on the top surface and aligns with the other electrode, of the same pair, located on the bottom surface. In this device, microparticles experience nDEP in the vertical and horizontal directions to focus the same at the center of the microchannel. Alnaimat et al. [10] developed the mathematical model of a microfluidic device with multiple pairs of IDT electrodes on both sides of the bottom surface of the microchannel for DEP-based 3D-focusing. The analyzed microfluidic device can 3D-focus microparticles at any lateral location along the width of the microchannel; for equal applied voltages, the microparticle is focused at the center of the microchannel and for unequal applied voltages the microparticle is focused at locations other than the center of the microchannel. Alnaimat et al. [16] also modeled a microfluidic device with two continuous electrodes each on the top and bottom surfaces of the microchannel; the electrodes on each side formed a pair. The minima of the gradient of the electric field is located at the center of the microchannel when both pairs of electrodes have equal applied voltages, thereby allowing for 3D-focusing of microparticles at the same. On the other hand, for unequal applied voltages the minima of the gradient of the electric field is located away from the center of the microchannel thereby allowing 3D-focusing microparticles at locations other than the center of the microchannel, specifically at the location of the minimum gradient of electric field.

Several electrode configurations have been developed in the past for DEP-based focusing [4]; however, most do not work well for microparticles very close to the sidewalls and can handle only low throughout. As already detailed, the electrode configuration presented in this article can overcome these drawbacks. Regarding the model developed in this article, it is a dynamic model, thereby allowing for determining the axial distance as well as duration required for reaching steady state conditions unlike static models. Knowledge of the axial distance required for reaching steady state dictates the actual size of the microfluidic device.

## 2. Theoretical Model

The model corresponding to the microfluidic device shown in Figure 2 is developed in this section. The purpose of the model is to calculate the trajectory of the microparticle in the microfluidic device and in turn determine the vertical and horizontal focusing parameters. The trajectory of the microparticle is described by Equation (4); it is based on Newton’s 2nd law and relates the acceleration of the microparticle to the net external force acting on the microparticle. External forces acting on the microparticle are drag, gravity, virtual mass, buoyancy, and DEP [10,16,17].

(4) $$\left[\begin{array}{c} {\ddot{x}}_{p}\\ {\ddot{y}}_{p}\\ {\ddot{z}}_{p} \end{array}\right]+9\frac{\mu_{m}}{\left(2\rho_{p}+\rho_{m}\right)r_{p}^{2}}\left[\begin{array}{c} {\dot{x}}_{p}\\ {\dot{y}}_{p}\\ {\dot{z}}_{p} \end{array}\right]-\frac{\rho_{m}}{\left(2\rho_{p}+\rho_{m}\right)}\left[\begin{array}{c} {\dot{u}}_{m,x}\\ {\dot{u}}_{m,y}\\ {\dot{u}}_{m,z} \end{array}\right]-9\frac{\mu_{m}}{\left(2\rho_{p}+\rho_{m}\right)r_{p}^{2}}\left[\begin{array}{c} u_{m,x}\\ u_{m,y}\\ u_{m,z} \end{array}\right]+2\frac{\left(\rho_{p}-\rho_{m}\right)}{\left(2\rho_{p}+\rho_{m}\right)}\left[\begin{array}{c} 0\\ 0\\ g \end{array}\right]-\frac{3}{2}\frac{\varepsilon_{m}Re\left[f_{CM}\right]}{\left(2\rho_{p}+\rho_{m}\right)}\left[\begin{array}{c} \frac{\partial }{\partial x}\\ \frac{\partial }{\partial y}\\ \frac{\partial }{\partial z} \end{array}\right]E_{RMS}^{2}=0$$

The initial conditions associated with the Equation (4) include the initial displacements and velocities as represented in Equations (5) and (6), respectively [10,16,17]. The microparticle can occupy any location along the cross-section of the microchannel at the start of the electrode configuration and this is presented in Equation (5). Moreover, it is medium that is dragging the microparticle to the start of the electrode configuration due to which the velocity of the microparticle at the start of the electrode configuration is same as that of the medium as shown in Equation (6).

(5) $${\left[\begin{array}{c} x_{p}\\ y_{p}\\ z_{p} \end{array}\right]|}^{t=0}=\left[\begin{array}{c} 0\\ Y\\ Z \end{array}\right]$$

(6) $${\left[\begin{array}{c} {\dot{x}}_{p}\\ {\dot{y}}_{p}\\ {\dot{z}}_{p} \end{array}\right]|}^{t=0}=\left[\begin{array}{c} {u_{m,x}|}_{x_{p}=0,y_{p}=Y,z_{p}=Z}\\ {u_{m,y}|}_{x_{p}=0,y_{p}=Y,z_{p}=Z}\\ {u_{m,z}|}_{x_{p}=0,y_{p}=Y,z_{p}=Z} \end{array}\right]$$

From Equation (4) it is evident that information on the electric field and velocity of the medium are required for solving the same. The information on electric field can be determined only if information on electric potential inside the microchannel is available. Information on electric potential inside the microchannel will be available upon solving Equation (7). Afterwards, solving Equation (8) will provide information on electric field inside the microchannel [10,16,17].

(7) $$\left(\frac{\partial^{2}}{\partial x^{2}}+\frac{\partial^{2}}{\partial y^{2}}+\frac{\partial^{2}}{\partial z^{2}}\right)V=0$$

(8) $$\left[\begin{array}{c} E_{x}\\ E_{y}\\ E_{z} \end{array}\right]=-\left[\begin{array}{c} \frac{\partial }{\partial x}\\ \frac{\partial }{\partial y}\\ \frac{\partial }{\partial z} \end{array}\right]V$$

The boundary conditions associated with Equation (7) include applied electric potential on the electrodes and electrical insulation elsewhere. All governing equations, i.e., Equations (4), (7) and (8), are numerically solved. The finite difference method (FDM) is used for solving Equations (4) and (7). For implementing FDM, second order central difference scheme replaces the first order differential term of Equation (4) while the second order central difference scheme of Equations (4) and (7) replaces second order central differential term. In order to evaluate the electric field, the first order differential terms of Equation (8) are replaced by second order difference schemes. The time step for Equation (4) is held at 0.00001 s. The node-to-node distance in the x-and z-directions are set at 25 μm and 1 μm, respectively. The node-to-node distance in the y-direction is determined based on the node-to-node in the x-direction and the electrode dimensions; this approach allows for having a structured grid inside the computational domain even though the electrode has a triangular shape. The details of implementing FDM to solve Equations (7) and (8) can be found in Alnaimat et al. [10,16] and Mathew et al. [17]. The implementation of FDM in Equation (4) and its subsequent rearrangement results in Equation (9) which can be used for finding displacements at the next time step. Equation (9) is evaluated until the axial displacement of the microparticle becomes equal to the length of the microfluidic device.

(9) $$\begin{array}{c} \left[\begin{array}{c} x_{p}^{n+1}\\ y_{p}^{n+1}\\ z_{p}^{n+1} \end{array}\right]=\left(\frac{1}{\frac{1}{\Delta t^{2}}+\frac{9}{2\Delta t}\frac{\mu_{m}}{\left(2\rho_{p}+\rho_{m}\right)r_{p}^{2}}}\right)(\frac{2}{\Delta t^{2}}\left[\begin{array}{c} x_{p}^{n}\\ y_{p}^{n}\\ z_{p}^{n} \end{array}\right]-\left(\frac{1}{\Delta t^{2}}-\frac{9}{2\Delta t}\frac{\mu_{m}}{\left(2\rho_{p}+\rho_{m}\right)r_{p}^{2}}\right)\left[\begin{array}{c} x_{p}^{n-1}\\ y_{p}^{n-1}\\ z_{p}^{n-1} \end{array}\right]+\frac{\rho_{m}}{\left(2\rho_{p}+\rho_{m}\right)}\left[\begin{array}{c} {\dot{u}}_{m,x}\\ {\dot{u}}_{m,y}\\ {\dot{u}}_{m,z} \end{array}\right]+\\ 9\frac{\mu_{m}}{\left(2\rho_{p}+\rho_{m}\right)r_{p}^{2}}\left[\begin{array}{c} u_{m,x}\\ u_{m,y}\\ u_{m,z} \end{array}\right]-2\frac{\left(\rho_{p}-\rho_{m}\right)}{\left(2\rho_{p}+\rho_{m}\right)}\left[\begin{array}{c} 0\\ 0\\ g \end{array}\right]+\frac{3}{2}\frac{\varepsilon_{m}Re\left[f_{CM}\right]}{\left(2\rho_{p}+\rho_{m}\right)}\left[\begin{array}{c} \frac{\partial }{\partial x}\\ \frac{\partial }{\partial y}\\ \frac{\partial }{\partial z} \end{array}\right]E_{RMS}^{2}) \end{array}$$

The velocity of the medium is dictated by the Navier–Stokes equations and the continuity equation. Additionally, the flow is usually fully developed at the start of the electrode configuration and this allows for reducing the governing equations to a simplified version of one of the components of the Navier–Stokes equation. The velocity of flow under these conditions is provided in Equation (10) [7]. Since the flow is fully developed, the medium has velocity only in the axial direction as stated in Equation (10).

(10) $$\left[\begin{array}{c} u_{m,x}\\ u_{m,y}\\ u_{m,z} \end{array}\right]=\left[\frac{48Q_{m}\sum_{i=1,3,5}^{\infty }\left(\frac{{\left(-1\right)}^{\left(\frac{i-1}{2}\right)}}{i^{3}}\right)cos\left[i\frac{\pi }{W_{ch}}\left(\frac{W_{ch}}{2}-y\right)\right]\left\{1-\frac{cosh\left[i\frac{\pi }{W_{ch}}\left(\frac{H_{ch}}{2}-z\right)\right]}{\cosh\left(\frac{i\pi }{2}\frac{H_{ch}}{W_{ch}}\right)}\right\}}{\begin{array}{c} \pi^{3}W_{ch}H_{ch}\left[1-\frac{192W_{ch}}{\pi^{5}H_{ch}}\sum_{i=1,3,5}^{\infty }\frac{tanh\left(i\frac{\pi }{2}\frac{H_{ch}}{W_{ch}}\right)}{i^{5}}\right]\\ 0\\ 0 \end{array}}\right]$$

The performance of the microfluidic device used for 3D-focusing is quantified in terms of Equations (11) and (12). Equations (11) and (12) represent the degree of 3D-focusing of microparticles. Mathematically, Equations (11) and (12) represent the standard deviation associated with the final position of the microparticle from the ideal 3D-focused position of the microparticle; performance of the microfluidic device studied by Morgan et al. [14] for 3D-focusing was quantified in a similar manner. Based on the working of the microfluidic device detailed above, the ideal 3D-focused position of the microparticle is the center of the microparticle. Reduction in magnitude of the vertical and horizontal focusing parameters indicates the closeness of the microparticles to the horizontal and vertical planes passing through the center of the microchannel, respectively. It is stressed here that smaller the focusing parameters, better the performance of the microfluidic device in achieving 3D-focusing. Both focusing parameters need to be small for the proposed device to realize 3D-focusing.

(11) $$\Delta w=\sqrt{\frac{\sum_{i=1}^{N}{\left(y_{p,i}-\frac{W_{ch}}{2}\right)}^{2}}{N-1}}$$

(12) $$\Delta d=\sqrt{\frac{\sum_{i=1}^{N}{\left(z_{p,i}-\frac{H_{ch}}{2}\right)}^{2}}{N-1}}$$

## 3. Model Validation

**Figure 4:**
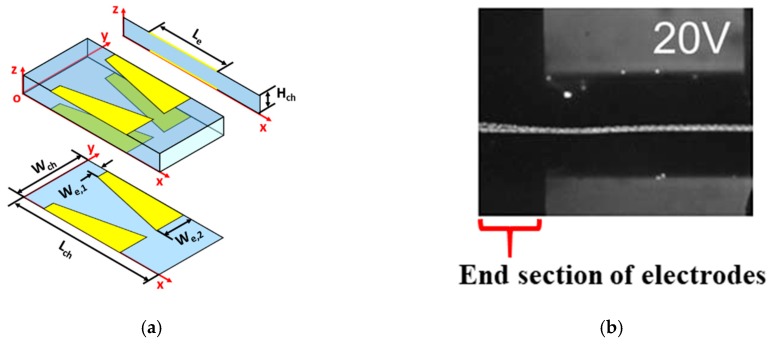

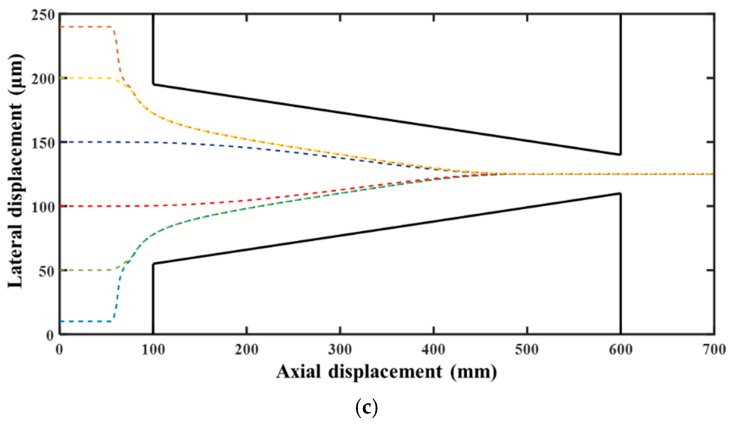
(**a**) Microfluidic device of Holmes et al. [9], (**b**) trajectories in the microfluidic device of Holmes et al. [9], and (**c**) trajectories based on the model (*r_p_* = 6 μm, *W_ch_* = 250 μm, *H_ch_* = 40 μm, *L_e_* = 500 μm, *L_ch_* = 700 μm, *W*_e,1_ = 55 μm, *W*_e,2_ = 105 μm). Figure 4b is reproduced with permission from Elsevier, Copyright (2006) from Holmes et al. [9].

Holmes et al. [9] developed a microfluidic device with two trapezoid shaped electrodes located on the top and bottom surfaces of the microchannel; the device is schematically shown in Figure 4. It can be noticed that the electrode configuration of Holmes et al. [9] is similar to that considered in this article; the difference being the non-zero starting width for the electrode configuration of Holmes et al. [9]. Holmes et al. [9] provided trajectories of microparticles in their device and this information is used for validating the model developed in this article. The width and height of the microchannel of the microfluidic device constructed by Holmes et al. [9] is 250 and 40 μm, respectively; the electrode length is 500 μm with the starting and ending widths of the electrodes being 55 and 105 μm, respectively. Holmes et al. [9] conducted tests for different applied electric potentials (0, 5, 10, 15, and 20 V_pp_) and documented the trajectories for all electric potentials. Holmes et al. [9] conducted tests using 6 μm (diameter) latex beads suspended in water. Information on volumetric flow rate (s) is not available in Holmes et al. [9]; however, they mention that for applied electric potential of 20 V_pp_ the axial velocity of microparticles is 1.05 mm/s after achieving cent percent 3D-focusing. For applied electric potential of 20 V_pp_, the microparticles are 3D-focused near the center of the microchannel and this coupled with the fact the microparticles are transported axially by the medium allows for assuming the centerline velocity of the medium to be same as the velocity of the microparticles. Based on the assumption that the centerline velocity of the medium to be 1.05 mm/s, the volumetric flow rate is determined and employed in the model. Figure 4 compares the trajectories of Holmes et al. [9] and the model; Holmes et al. [9] provided information on the trajectories at the end of the electrodes as shown in Figure 4. The model is used to calculate the trajectory of microparticles starting from several locations along the width of the microchannel as depicted in Figure 4. Upon comparison of the trajectories, it can be noticed that there is good match between the two and this validates the model. 

The model detailed here is based on several assumptions all of which are detailed in Mathew et al. [18] and includes negligible interaction between microparticles as well as between the microparticle and medium. These assumptions are satisfactorily achieved in all microfluidic devices handling dilute samples. On the other hand, when handling dense suspensions these assumptions do not hold leading to the need for modifying the coefficient associated with the drag equation. Knoerzer et al. [19] developed an algorithm, called dynamic drag force based on iterative density mapping, for determining the trajectory of microparticles while including the effect of these interactions; their model has been experimentally validated.

## 4. Results and Discussions

**Figure 5:**
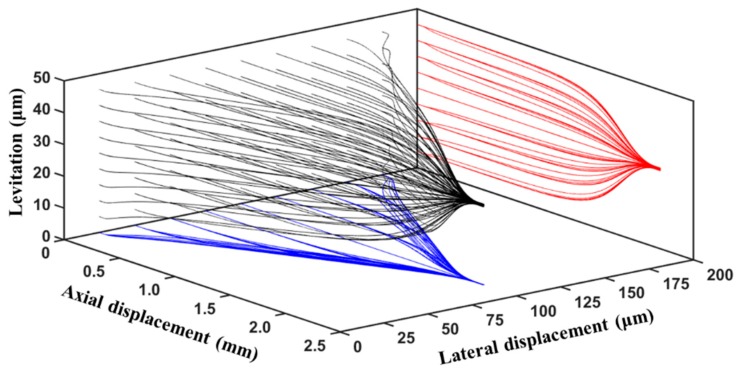
Trajectories of microparticles in the microfluidic device subjected to negative-DEP (nDEP) force (*r_p_* = 2 μm, *V*_1_ = *V*_2_ = 20 V_pp_, *L_e_* = 2000 μm, *L_ch_* = 2200 μm, *W_e_* = 80 μm, *Q_m_* = 100 μL/h, *W_ch_* = 200 μm, *H_ch_* = 50 μm).

The ability of the microfluidic device in achieving 3D-focusing is investigated in this section. Studies are carried out using polystyrene microparticles suspended in water (*ε_m_* = 78.5*ε_o_*, *ρ*_m_ = 998 kg/m^3^, *μ_m_* = 10^−3^ Pa·s) [8]. The trajectories of 81 microparticles (uniformly distributed) originating from the inlet of the microchannel are shown in Figure 5 along with projections of each trajectory on to vertical and horizontal planes; similar simulations with 100 microparticles were carried out by Morgan et al. [9]. It can be noticed that the proposed microfluidic device can achieve 3D-focusing at the center of the microchannel irrespective of the initial position of the microparticle. For this electrode configuration, the horizontal DEP force in the vertical plane passing through the center of the microchannel is zero and this leads to all microparticles being horizontally pushed to this vertical plane. Similarly, the vertical component of the nDEP force is zero in the horizontal plane passing through the center of the microchannel which causes all microparticles to experience vertical motion towards this horizontal plane. The vertical and horizontal motions of the microparticles happen simultaneously, thereby leading them to be almost 3D-focused at the center of the microchannel.

From the equations presented in the previous section of the article it is evident that the performance of the microfluidic device depends on parameters such as microchannel height, electrode width and length, volumetric flow rate, and applied electric potential. In the following part of this article, the influence of these parameters are investigated.

**Figure 6:**
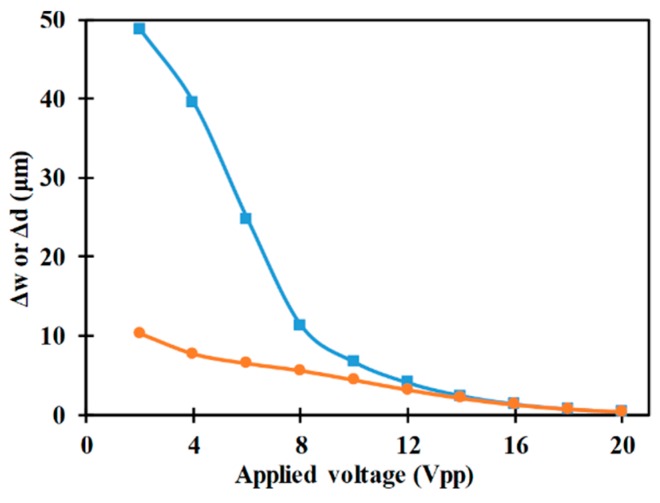
Variation of Δw (■) and Δd (●) with applied voltage (*r_p_* = 2 μm, *L_e_* = 2000 μm, *L_ch_* = 2200 μm, *W_e_* = 80 μm, *Q_m_* = 100 μl/h, *W_ch_* = 200 μm, *H_ch_* = 50 μm).

Figure 6 presents the variation of horizontal and vertical focusing parameters with respect to the applied electric potential. It can be noticed that with increase in applied electric potential the focusing parameters decrease indicating the enhancement in performance of the microfluidic device in achieving 3D-focusing. An increase in applied electric potential increases the electric potential inside the microchannel which in turn increases all components of the nDEP force inside the microchannel. This increase in components of the nDEP force causes the microparticles to move closer to the horizontal and vertical planes passing through the center of the microchannel, while passing through the region of the electrodes, thereby leading to the observed increase in performance of the microfluidic device. At low values of applied electric potential, the focusing parameters is almost equal to the focusing parameters in the absence of applied electric potential. The horizontal and vertical focusing parameters in the absence of applied electric potential are ca. 52 and ca. 13 μm, respectively. This indicates that there exists a threshold applied electric potential below which no appreciable degree of focusing can be expected when all other parameters are maintained constant.

Additionally, it can also be noticed from Figure 6 that the vertical focusing parameter is significantly smaller than the horizontal focusing parameter for low applied electric potentials. The width of the microchannel is much greater than the height of the microchannel; the small height implies that the electric field, and subsequently the nDEP force, is stronger along the height than along the width. This, coupled with the fact that the average displacement required to reach the horizontal plane passing through the center of the microchannel is smaller than the average displacement required to reach the vertical plane passing through the center of the microchannel, leads to vertical focusing parameter being smaller than horizontal focusing parameter. At high applied voltages, the electric field and the nDEP forces are strong in both directions to realize excellent degree of focusing in the vertical and horizontal directions thereby leading to both focusing parameters assuming low values.

**Figure 7:**
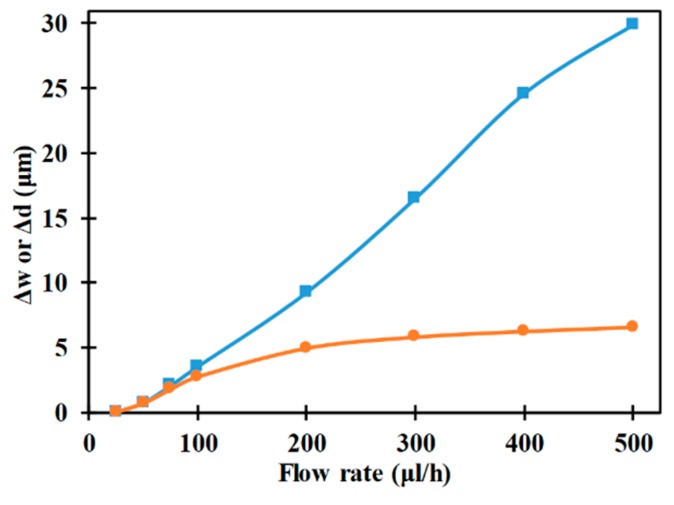
Variation of Δw (■) and Δd (●) with flow rare (*r_p_* = 2 μm, *L_e_* = 2000 μm, *L_ch_* = 2200 μm, *W_e_* = 80 μm, *V*_1_ = *V*_2_ = 12 V_pp_, *W_ch_* = 200 μm, *H_ch_* = 50 μm).

Figure 7 shows the influence of volumetric flow rate on the horizontal and vertical focusing parameters. Increase in volumetric flow rate increases the horizontal focusing parameter indicating degradation in the ability of the microfluidic device to achieve 3D-focuisng. Increase in volumetric flow rate increases the x-component of the force associated with drag at every location inside the microchannel while maintaining the x-component of the nDEP force constant. Thus, with an increase in volumetric flow rate, the velocity of the microparticle in the axial direction increases. An increase in velocity of the microparticle in the axial direction reduces the duration for which the same is subjected to y-component of nDEP force. This reduction in duration leads to deterioration of the degree of deflection of the microparticle towards the interior of the microchannel which ultimately causes the microparticle to move into the region between the top and bottom electrodes. Once the microparticle moves into this space, between the electrodes, it experiences weakening nDEP force due to which there is no further chance of being focused in the horizontal direction. Increase in volumetric flow rate increases the vertical focusing parameter as well for the same reason, i.e. reduction in residence time for which the *z*-component of nDEP force acts on the microparticle. It can be noticed form Figure 7 that the variation of the vertical focusing parameter with volumetric flow rate is much smaller than that of horizontal focusing parameter with respect to volumetric flow rate and it is due to the fact that the width of the microchannel is much greater than the height of the microchannel. Additionally, it can be concluded from Figure 7 that an increase in volumetric flow rate will cause the focusing parameters to increase till they become equal to the focusing parameters of a device without any focusing ability, i.e., horizontal and vertical focusing parameters of ca. 52 μm and ca. 13 μm, respectively. This indicates that there is an upper limit for volumetric flow rate beyond which no focusing is possible when all other parameters are maintained constant.

**Figure 8:**
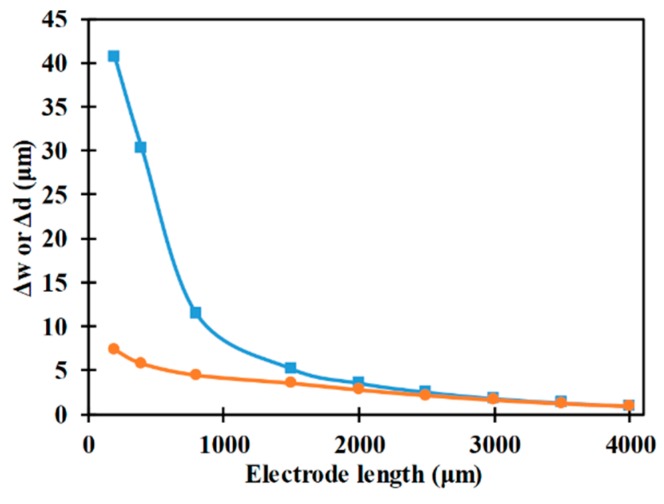
Variation of Δw (■) and Δd (●) with electrode length (*r_p_* = 2 μm, *W_e_* = 80 μm, *L_ch_* = *L_e_* + 200 μm, *V*_1_ = *V*_2_ = 12 V_pp_, *W_ch_* = 200 μm, *H_ch_* = 50 μm).

Figure 8 represents the influence of electrode length on the vertical and horizontal focusing parameters. It can be noticed that with an increase in electrode length the horizontal focusing parameter reduces which in turn depict enhancement in performance of the microfluidic device in achieving 3D-focusing. An increase in length of the electrodes reduces the orientation of the electrodes, with respect to the sidewalls, which in turn enhances the y-component of the nDEP force acting on the microparticle. Additionally, an increase in electrode length reduces the x-component of the nDEP force which along with the x-component of the drag remaining constant leads to an increase in velocity of the microparticle. The increase in the y-component of the nDEP force with the increase in electrode length is greater than the increase in velocity of the microparticle, in the axial direction, under the same conditions and these lead to the reduction in horizontal focusing parameter as observed in Figure 7. Regarding the vertical focusing parameter, it reduces with the increase in electrode length as well and this can be attributed to the increase in the residence time for which the microparticles are subjected to the nDEP force in the vertical direction. Additionally, it can be noticed that the variation of the vertical focusing parameter with electrode length is smaller than that associated with horizontal focusing parameter and it is due to the microchannel height being smaller than the microchannel width.

**Figure 9:**
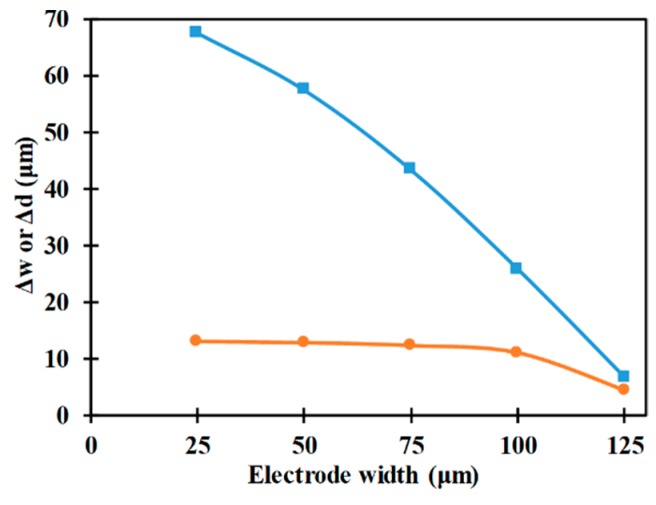
Variation of Δw (■) and Δd (●) with electrode width (*r_p_* = 2 μm, *L_e_* = 2000 μm, *L_ch_* = 2200 μm, *V*_1_ = *V*_2_ = 12 V_pp_, *W_ch_* = 200 μm, *H_ch_* = 50 μm).

The influence of the electrode width on the vertical and horizontal focusing parameters is analyzed in Figure 9; focusing parameters decrease with the increase in electrode width, which is indicative of the improvement in the performance of the microfluidic device in achieving 3D-focusing. Increase in electrode width increases the lateral displacement of the microparticle towards the center of the microchannel which in turn leads to it ending up closer to the center of the microchannel after passing over the length of the electrodes. With an increase in electrode width, the orientation of the electrode with respect to the sidewall increases, thereby leading to increase and decrease in the x- and y-components of the nDEP force, respectively. The increase in the x-component of the nDEP forces leads to reduction in the velocity of the microparticles in the axial direction. The reduction in the y-component of the nDEP force, with change in electrode width, is smaller than the reduction in the axial velocity, with change in electrode width, and this allows for the microparticle to be subjected to nDEP force for duration required to achieve the desired deflection.

Regarding the vertical focusing parameter, it remains almost constant with an increase in electrode width before significantly reducing at high electrode width; the vertical focusing parameters at low electrode widths is almost equal to the vertical focusing parameter in the absence of nDEP forces, i.e., ca. 13 μm. With an increase in electrode width the number of microparticles occupying the space between the top and bottom electrodes increase along with the increase in degree of non-uniformity of the electric field. These are the reasons for the observed trend in the variation of vertical focusing parameter with electrode width. Since microparticles are uniformly distributed over the cross-section of the microchannel, the distance between each sidewall and the nearest column of microparticles is 30 μm, while that between each sidewall and the nearest second and third columns of microparticles are 60 μm and 90 μm, respectively. Thus, for electrode width of 25 μm no microparticles are present in the space between the top and bottom electrodes while for electrode widths of 50 µm and 75 µm the number of columns of microparticles present in the space between the electrodes are one and two, respectively. For an electrode width of 125 µm, the first four columns of microparticles from each sidewall occupy the space between the electrodes and this coupled with enhancement z-component of nDEP force, associated with the increase in electrode width, leads to strong vertical focusing as depicted in Figure 9.

**Figure 10:**
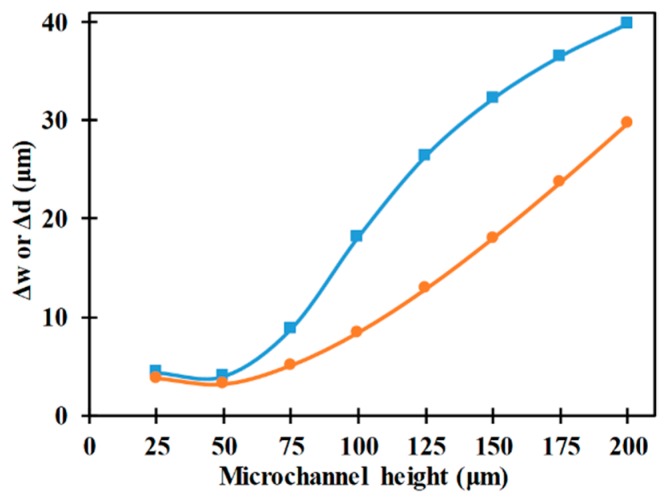
Variation of Δw (■) and Δd (●) with electrode height (*r_p_* = 2 μm, *L_e_* = 2000 μm, *L_ch_* = 2200 μm, *W_e_* = 80 μm, *V*_1_ = *V*_2_ = 12 V_pp_, *W_ch_* = 200 μm).

Figure 10 shows the variation of focusing parameters with respect to microchannel height. It can be noticed that an increase in microchannel height initially decreases the focusing parameters before increasing them. This indicates the existence of an optimal microchannel height for purposes of 3D-focusing using the electrode configuration shown in Figure 2. The initial increase in microchannel height leads to an increase in non-uniformity of the electric field inside the microchannel and this leads to an increase in the nDEP force in all directions thereby leading to the decrease in focusing parameters. On the other hand, with a further increase in microchannel height the electric field inside the microchannel decreases leading to the reduction of the nDEP force in all directions which in turn leads to the degradation in performance of the microfluidic device, i.e., increase in focusing parameters. It can also be noticed that as the microchannel height becomes equal to the microchannel width, the focusing parameters become comparable; this is due to the nDEP forces in both directions becoming comparable.

**Figure 11:**
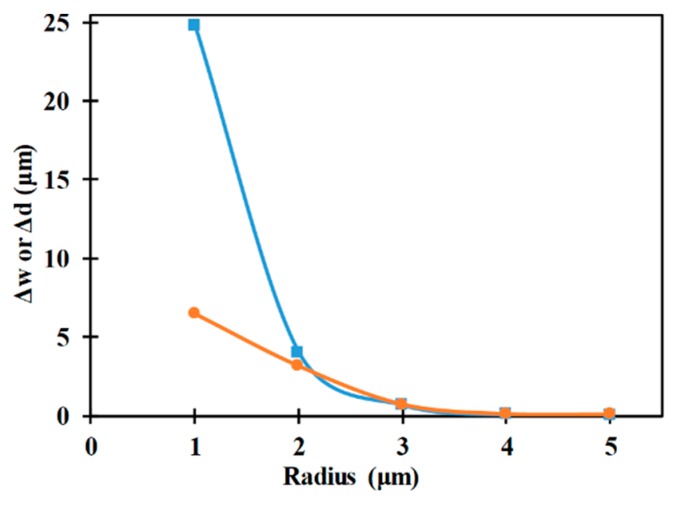
Variation of Δw (■) and Δd (●) with microparticle radius (*W_e_* = 80 μm, *L_e_* = 2000 μm, *L_ch_* = 2200 μm, *V*_1_ = *V*_2_ = 12 V_pp_, *W_ch_* = 200 μm, *H_ch_* = 50 μm).

Figure 11 depicts the influence of microparticle radius on the vertical and horizontal focusing parameters associated with the microfluidic device. It can be noticed that increase in microparticle radius reduces the focusing parameters which in turn indicates that enhancement in performancve of the microfluidic device. This is because the nDEP force depends on the cubic power of the radius, as can be observed from Equation (1).

## 5. Conclusions

This article presents a nozzle-shaped electrode configuration for dielectrophoresis-based 3D-focusing of microparticles. A mathematical model of this microfluidic device is developed and used for studying its feasibility in achieving 3D-focusing. Additionally, the mathematical model is used for parametric study to understand the influence of operating/geometric parameters on the 3D-focusing efficiency of the device. The parameters analyzed include applied electric potential, volumetric flow rate, electrode width and length, and microchannel height. The performance of the microfluidic device is quantified in terms of horizontal and vertical focusing parameters which are mathematically equivalent to the standard deviation of the horizontal and vertical positions of the microparticle evaluated at the exit section on the microfluidic device. The focusing parameters decrease with an increase in applied electric potential, thereby indicating enhancement in performance of the microfluidic device. The focusing parameters increase with an increase in volumetric flow rate, which is indicative of the degradation in performance of the microfluidic device. An increase in electrode width and length improves the focusing parameters, thereby indicating the enhancement in performance of the microfluidic device. An increase in microchannel height generally increases the focusing parameters, thereby leading to degradation in performance of the microfluidic device. The focusing parameters are influenced by the radius of the microparticle such that an increase in radius of microparticle reduces the focusing parameters, thereby indicating improvement in the performance of the microfluidic device. The model is particularly useful in selecting the geometric and operating parameters of the proposed microfluidic device so as to operate it at the desired performance metrics, i.e., focusing parameters.

## Nomenclature

*Δd*	*horizontal focusing parameter (m or μm)*
*E*	*electric field (V/m)*
*F*	*force (N)*
*H*	*height (m or μm)*
*K_s_*	*surface conductance (S)*
*N*	*number of microparticles considered for determining focusing parameters*
*Re[f_CM_]*	*real part of Clausius–Mossotti factor*
*r*	*radius (m or μm)*
*u*	*velocity (m/s)*
*V*	*applied electric voltage (V)*
*W*	*width (m or μm)*
*Δw*	*vertical focusing parameter (m or μm)*
*x*	*displacement in the x-direction (m or μm)*
*Y*	*initial position of microparticles along the width of the microchannel (m or μm)*
*y*	*displacement in the y-direction (m or μm)*
*Z*	*initial positon of microparticle along the height of the microchannel (m or μm)*
*z*	*displacement in the z-direction (m or μm)*
**Greek alphabet**	
Ε	permittivity (F/m)
ε_o_	permittivity of free space (F/m)
Σ	conductivity (S/m)
*Ρ*	density (kg/m^3^)
Ω	operating frequency (rad/s)
**Subscripts**	
1	*electrode pair on the right-hand side with respect to the flow direction*
2	*electrode pair on the left-hand side with respect to the flow direction*
*bulk*	*bulk*
*DEP*	*dielectrophoresis*
*m*	*medium*
*p*	*microparticle*
